# Repeated Systolic Anterior Motion of the Mitral Valve After Double Outlet Right Ventricle Repair

**DOI:** 10.1016/j.atssr.2024.09.003

**Published:** 2024-09-26

**Authors:** Junya Sugiura, Hajime Sakurai, Wataru Kato, Keisuke Tanaka, Koji Morita, Koshi Yamaki, Taisei Nagashima

**Affiliations:** 1 Department of Cardiovascular Surgery, Japan Red Cross Aichi Medical Center Nagoya Daini Hospital, Nagoya, Japan; 2 Department of Cardiac Surgery, Nagoya University Graduate School of Medicine, Nagoya, Japan

## Abstract

We herein report a rare case of double outlet right ventricle repair complicated by repeated systolic anterior motion of the mitral valve after weaning from cardiopulmonary bypass and postoperatively. Even in patients without obvious preoperative left ventricular outflow tract obstruction, systolic anterior motion and mitral regurgitation may still occur from functional factors, such as intraoperative or postoperative inotropic drug use, tachycardia, or intraventricular volume loss in patients with severe ventricular septal hypertrophy and left ventricular hypertrophy.

Systolic anterior motion (SAM) of the anterior leaflet of the mitral valve can be observed in cases of left ventricular outflow tract (LVOT) obstruction, including hypertrophic obstructive cardiomyopathy (HOCM) and after mitral valvuloplasty. SAM may require re-repair or replacement of mitral valve, especially after mitral valvuloplasty.^1^ Although there have been several reports of septal myectomy for HOCM in children,^2^^,^^3^ it is rare to find a case of SAM and mitral regurgitation (MR) after intracardiac repair of congenital heart disease in pediatric cases in which this was not a problem before operation. Here, we describe a case of SAM and severe MR after weaning from cardiopulmonary bypass following repair of double outlet right ventricle (DORV) with ventricular hypertrophy.

A 10-month-old girl with DORV of subaortic type ventricular septal defect and biventricular hypertrophy underwent pulmonary artery banding at 25 days of age as primary surgery. Progressive cyanosis was observed at approximately 6 months of age, and cardiac catheterization showed a Qp/Qs ratio of 1.05, Rp index 1.31 Wood units·m^2^, right ventricle (apex) pressure of 104/12 mm Hg, and left ventricle pressure of 108/13 mm Hg. Echocardiography showed progressive biventricular hypertrophy, with a ventricular septum of 13 mm and a left ventricular posterior wall of 8 mm (ratio, 1.6 [>1.3]), meeting the criteria for asymmetric ventricular septal hypertrophy, no obvious LVOT obstruction, and trivial MR (Figure 1).

**Figure 1:**
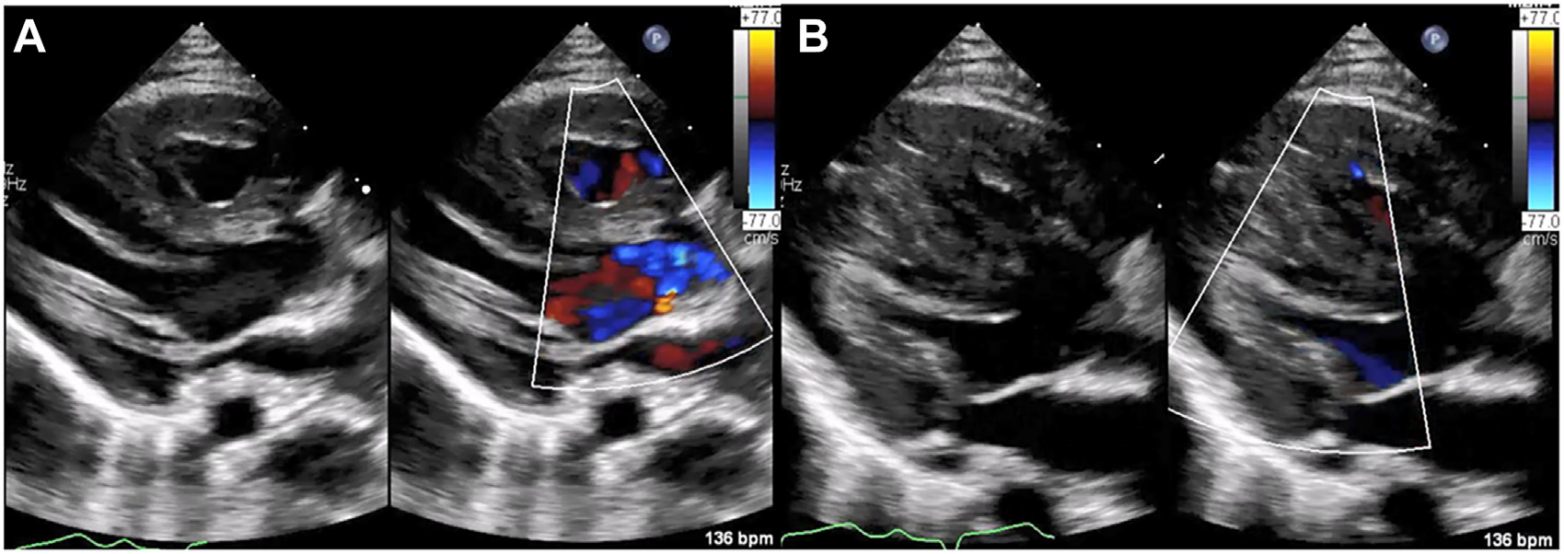
(A, B) Preoperative transthoracic echocardiography showing a hypertrophic biventricle and ventricular septum directing to the mitral valve, meeting the criteria for asymmetric ventricular septal hypertrophy.

The patient underwent intracardiac DORV repair at 10 months of age. After secondary median sternotomy and standard central aortic and bicaval venous cannulation, cardiopulmonary bypass was initiated. After aortic cross-clamping, the main pulmonary artery was dissected at the pulmonary artery banding site, an anterior right ventricular outflow tract (RVOT) incision was made, right ventricular myectomy was performed, and the patient underwent pathologic examination for possible hypertrophic cardiomyopathy or secondary cardiomyopathy. The perimembranous outlet ventricular septal defect was closed with a 0.4-mm Gore-Tex patch (W. L. Gore & Associates). After aortic declamping, the RVOT was closed with a 0.6-mm Gore-Tex patch, and the main pulmonary artery was repaired with end-to-end anastomosis. After weaning from cardiopulmonary bypass, pressure measurements showed a right ventricle (inflow) pressure of 41/12 mm Hg and central aortic pressure of 89/46 mm Hg. When inotropic drugs seemed to be effective, the heart rate (HR) increased to 160 beats/min and systolic blood pressure dropped to 50 mm Hg. At this time, transesophageal echocardiography showed severe MR, and the anterior leaflet of the mitral valve was stuck at the LVOT in both the systolic and diastolic phases, indicating SAM (Figure 2). In response, volume loading and reduction in inotropic drugs resulted in a decrease in HR and an increase in systolic blood pressure. Transesophageal echocardiography showed an improvement in MR to trivial values. The sternum was left open.

**Figure 2:**
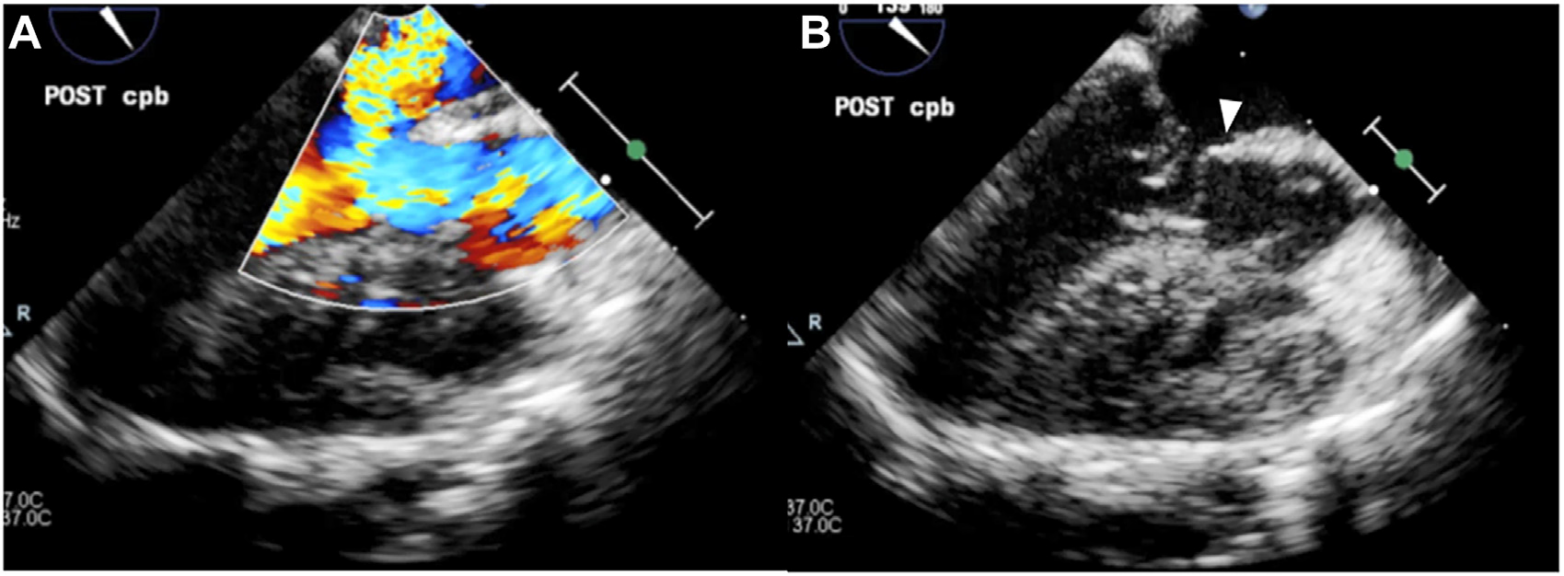
Intraoperative transesophageal echocardiography showed (A) severe mitral regurgitation and (B) systolic anterior motion of anterior leaflet of mitral valve after weaning from cardiopulmonary bypass (cpb; arrowhead).

Postoperatively, deep sedation and cooling were performed to maintain the HR as low as possible. On postoperative day 1, the HR decreased to 120 beats/min, and the MR disappeared. On postoperative day 6, the sternum was closed; however, on postoperative days 4 to 6, recurrence of SAM and MR up to a moderate degree was repeatedly observed, along with HR elevation due to fever increase or awakening, which required antifebrile agents and beta blockers. On postoperative day 7, the HR was no longer elevated, even after further awakening, following stabilization of the MR from none to trivial. The patient was extubated on postoperative day 10 and discharged on postoperative day 22.

Predischarge echocardiography showed trivial MR, no LVOT obstruction (1.0 m/s), and no RVOT obstruction. Pathologic examination revealed mild to moderate hypertrophy of myocardial cells with an intricate array. Perivascular and interstitial fibrosis was observed, suggesting the possibility of hypertrophic cardiomyopathy. No images suggested Fabry or Danon disease. The result of genetic testing for Noonan syndrome was negative.

## Comment

The cause of SAM has traditionally been thought to be the Venturi effect, induced by the high-flow velocity from the LVOT obstruction in HOCM, which pulls the anterior leaflet of the mitral valve toward the ventricular septum. However, it is now believed that HOCM causes anterior deviation of the papillary muscles, excess mitral valve tissue, and thickening of the midventricular septum, which redirects the outflow, and abnormally directed outflow gets behind the mitral valve, catches it, and pushes the anterior leaflet into the septum, explained by the pushing (drag) force.^4^^,^^5^

Functional causes of SAM include ventricular hypercontraction due to inotropic drug use, intraventricular volume loss immediately after weaning from cardiopulmonary bypass or by postoperative capillary leakage, and left ventricular diastolic dysfunction due to thickened myocardium or myocardial edema due to cardiac arrest.^1^ These functional factors may be important in considering responses to SAM events.

In this case, a postoperative increase in HR due to an increase in fever, awakening from sedation, and a hyperdynamic state of post-debanding of the main pulmonary artery were potential causes for the decrease in stroke volume. The reason for the halt in HR elevation on the day after sternal closure was thought to be intravascular refilling or improvement in sedation withdrawal symptoms.

As described by Crescenzi and colleagues^1^ in their article on SAM after mitral valvuloplasty, the first step in managing SAM is intravascular volume expansion and discontinuation of the inotropic drugs. The second step involves manual compression of the ascending aorta and the simultaneous administration of beta blockers. If SAM does not improve after these measures, surgical intervention should be considered. Right ventricular pacing may also be an option to improve dynamic LVOT obstruction.

**Figure 3:**
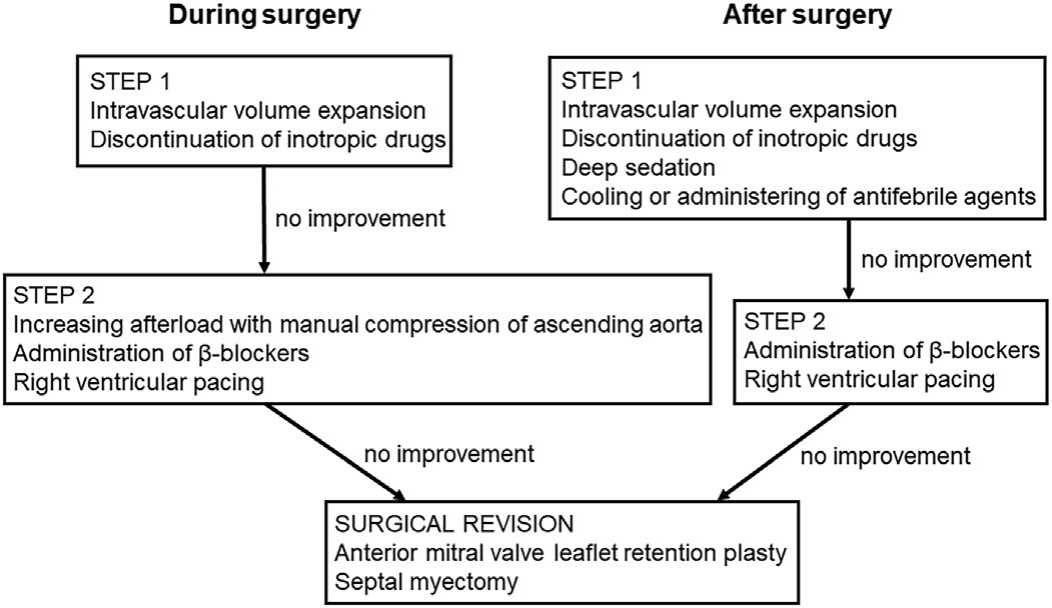
Management of the systolic anterior motion related to either dynamic or morphologic left ventricular outflow tract obstruction during or after surgery in children with congenital heart disease.

Unlike SAM after mitral valvuloplasty in adult patients, in this case, surgical intervention would require anterior mitral valve leaflet retention plasty or septal myectomy.^6^ However, the patient did not have morphologic LVOT obstruction despite significant preoperative ventricular septal hypertrophy. The SAM was thought to have been caused largely by functional factors and did not require surgical intervention (Figure 3). If myocardial edema due to cardiac arrest or a hyperdynamic state of post-debanding of the pulmonary artery was involved as a cause of SAM, improvement could be expected over time after surgery.

In conclusion, even in patients without obvious preoperative LVOT obstruction, SAM and MR may be due to functional factors such as intraoperative or postoperative inotropic drug use, tachycardia, or intraventricular volume loss in patients with severe ventricular septal hypertrophy and left ventricular hypertrophy.
